# ﻿A new species of Clematis sect. Tubulosae (Ranunculaceae) from Zhejiang, East China

**DOI:** 10.3897/phytokeys.267.158140

**Published:** 2025-12-03

**Authors:** Pan Xu, Jun-Ping Li, Xian-Ting Wang, Fen-Yao Zhang, Wei-Qing Liang, Wen-Yuan Xie, Li-Peng Yu, Feng Chen, Ke-Lang Lou, Jian-Ping Zhong, Jin-Bao Pu, Zheng-Hai Chen

**Affiliations:** 1 Center for Medicinal Resources Research, Zhejiang Academy of Traditional Chinese Medicine, Hangzhou 311300, Zhejiang, China Zhejiang Academy of Traditional Chinese Medicine Hangzhou China; 2 Zhejiang Engineering Research Center for Quality Assessment and Development of Dao-di Herbs, Hangzhou 311300, Zhejiang, China Zhejiang Engineering Research Center for Quality Assessment and Development of Dao-di Herbs Hangzhou China; 3 Yongkang Forestry Bureau, Yongkang 321300, Zhejiang, China Yongkang Forestry Bureau Yongkang China; 4 Zhejiang Hynobius amjiensis National Nature Reserve Management Center, Anji 313304, Zhejiang, China Zhejiang Hynobius amjiensis National Nature Reserve Management Center Anji China; 5 Zhejiang Forest Resources Monitoring Centre, Hangzhou 310020, Zhejiang, China Zhejiang Forest Resources Monitoring Centre Hangzhou China; 6 Zhejiang Forestry Survey Planning and Design Co. Ltd., Hangzhou 310020, Zhejiang, China Zhejiang Forestry Survey Planning and Design Co. Ltd. Hangzhou China

**Keywords:** *
Clematis
*, morphology, new species, phylogeny, plastid genome, taxonomy

## Abstract

*Clematis liana* sp. nov., a new species of Ranunculaceae from Zhejiang and Anhui Provinces in eastern China, is described and illustrated. This species exhibits similarities in morphological features to *C. urticifolia*, with which it has a close evolutionary relationship. It is distinguishable by several discrete features, such as habit, reproductive strategy, morphology of cauline ridges, leaflets, bracts, and flowers, calycine color, number of carpels and stamens, length and indumentum of filaments, size of anthers and pollen grains, etc. The complete plastid genome sequence of *C. liana***sp. nov.** comprises 159,759 bp, organized into a quadripartite structure containing two inverted repeat (IR: 31,084 bp each) regions, a small single copy (SSC: 18,135 bp) region, and a large single copy (LSC: 79,456 bp) region. It contains 136 functional, comprising 92 protein-coding, 36 tRNA and 8 rRNA genes.

## ﻿Introduction

Sect. Tubulosae Decne. (1881: 203) is classified within the Ranunculaceae family, under the genus *Clematis* L. (1753: 543), subgenus Clematis. This section is endemic to East Asia, as documented by Tamura (1995), Wang and Li (2005), Wang and Xie (2007), and Lyu et al. (2023). According to the most recent taxonomic treatment, sect. Tubulosae has been divided into two subsections: subsect. Pinnatae (W.T. Wang) W. T. Wang and subsect. Tubulosae (Decne.) W.T. Wang (Wang and Xie 2007). The latter comprises ten taxa (eight species and two varieties) distributed across the Japanese Archipelago, the Korean Peninsula, Taiwan and mainland China (Wang 2001, Wang and Li 2005, Kadota 2006, Wang and Xie 2007, Lyu et al. 2023). Among these, *C. heracleifolia* DC. (1818: 138), *C. tubulosa* Turcz. (1837: 148), and C. tubulosa var. ichangensis (Rehder and E.H. Wilson) W.T. Wang (2006: 335) are native to mainland China. *C. heracleifolia* and *C. tubulosa* are restricted to northern China, whereas C. tubulosa var. ichangensis occurs in southern part of subsect. Tubulosae, including Hubei, Henan, Hunan, Shaanxi, and Shanxi provinces, with scattered populations in Anhui, Zhejiang, and Guizhou (Wang 2001, Wang and Li 2005, Wang and Xie 2007, Zhang 2021, Lyu et al. 2023). The remaining seven taxa of sect. Tubulosae are narrow endemics found in the Korean Peninsula and adjacent East Asia. Among them, *C. urticifolia* Nakai ex Kitag. (1937: 346) is native to Korean, *C. stans* Siebold & Zucc. (1845: 177), C. stans var. austrojaponensis (Ohwi) Ohwi (1953: 513), *C. speciosa* (Makino) Makino (1918: 39) and *C. satomiana*Kadota (2006: 301) occur in the Japanese Archipelago (Wang 2001, Wang and Li 2005, Kadota 2006, Wang and Xie 2007, Lyu et al. 2023), while *C. psilandra* Kitag. (1937: 352) and *C. tsugetorum*Ohwi (1933: 153) are distributed in Taiwan (Yang and Huang 1996, Wang 2001, Wang and Bartholomew 2001, Wang and Li 2005, Wang and Xie 2007, Lyu et al. 2023).

In 2013, during a survey of wild plant resources, Professor Gen-You Li from Jiyang College Zhejiang A&F University, and Zheng-Hai Chen discovered an unknown *Clematis* taxon on Longwangshan moutain in Anji County, Huzhou City, Zhejiang Province, eastern China. Subsequently, targeted field investigation was conducted to collect more data.

The newly discovered plants are erect perennial herbs, characterized by urceolate flowers with four imbricate, erect sepals, creamy white or pale yellow, and narrowly ovate or lanceolate. The leaves are ternate. The pollen grains are pantoporate. Based on the current infrageneric classification derived from morphological traits (Wang and Li 2005, Wang and Xie 2007, Lyu et al. 2023), these plants are undoubtedly placed in subsect. Tubulosae, ser. Tubulosae Rehder & E.H. Wilson (1913: 320). Morphologically, they show the greatest similarity to *C. urticifolia* Nakai ex Kitag. (Kitagawa 1937; Wang and Xie 2007) from Korea. However, further research reveals considerable morphological distinctions between this newly found taxon and *C. urticifolia*, leading us to reassess its taxonomic identity.

## ﻿Materials and methods

### ﻿Morphological observation

From 2013 to 2024, we conducted more than ten field surveys in the hope of finding new populations of new discovered species in Anji County and its surrounding areas. For the three previously confirmed subpopulations of *C. liana* sp. nov. in Longwangshan mountain, we established fixed-location observation plots to document phenology, morphological structure, and ecological habits through quarterly monitoring of tagged plants. At the same time, specimens in the collections of most herbaria in Zhejiang Province (HHBG, HTC, HZU, ZJFC, and ZM) (Thiers 2024) and photos of specimens on CVH (https://www.cvh.ac.cn/) were checked to identify specimens similar to the unknown taxon. High-definition images of similar species-type specimens from foreign herbaria (A, BM, DAO, K, L, M, P, and PH) (Thiers 2024) were also consulted. Based on field investigation and herbarium specimen observation, the morphology of this new species was documented by comparing with the protologue of *C. urticifolia* (Kitagawa 1937).

### ﻿Taxon sampling

On 5 August and 10 November, 2024, we collected six individuals from two populations in Longwangshan mountain, Anji County, Huzhou City, and Shunxiwu, Lin’an District, Hangzhou City, Zhejiang Province, respectively (Table 1). The leaves from six individuals were first placed in non-woven bags, and then stored in sealing bags containing allochroic silica gel for DNA extraction. The voucher specimens are preserved in the Herbarium of Medicinal Resources, Zhejiang Academy of Traditional Chinese Medicine (ZJMR), and the Zhejiang Museum of Natural History (ZM).

**Table 1.** List of analyzed samples of *Clematis liana*.

Molecular specimen	Voucher specimen	Collection location	Longitude and latitude	Altitude(m)
AJ-01	TXLAJ2024080501	Dongguan to Xianrenqiao, Longwangshan mountain, Anji County, Huzhou City	30°24'25.55"N, 119°26'35.69"E	1,248
AJ-05	TXLAJ2024080505	Dongguan to Xianrenqiao, Longwangshan mountain, Anji County, Huzhou City	30°24'27.58"N, 119°26'28.71"E	1,173
AJ-06	TXLAJ2024080506	Dongguan to Xianrenqiao, Longwangshan mountain, Anji County, Huzhou City	30°24'29.46"N, 119°26'22.52"E	987
LA-01	*LATXL2024111001*	Taoshuwan, Shunxiwu, Linan district, Hangzhou City	30°1'10"N, 118°56'24"E	1,028
LA-02	*LATXL2024111002*	Taoshuwan, Shunxiwu, Linan district, Hangzhou City	30°1'10"N, 118°56'24"E	1,028
LA-03	*LATXL2024111003*	Taoshuwan, Shunxiwu, Linan district, Hangzhou City	30°1'10"N, 118°56'24"E	1,028

### ﻿DNA extraction, PCR amplification, and sequencing

Genomic DNA was extracted using a modified CTAB protocol following the instructions of Plant Genomic DNA Kit (Tiangen Biotech Co., Beijing, China). The total gDNA sample was sequenced by Tsingke Biotechnology Co. (China). For ribosomal DNA (rDNA) amplification, the ITS as primer (Forward: 5'- ATGCGATACTTGGTGTGAAT- 3', Reverse: 5'- GACGCTTCTCCAGACTACAAT - 3') (White et al. 1990). PCR reaction program proceeded according to Xie et al. (2011). Briefly, DNA amplifications were conducted in 25 μL reaction containing 10–50 ng of total DNA, 20 mmol/L Tris buffer (pH 8.3, with 50 mmol/L KCl, 1.5 mmol/L MgCl_2_, and 0.1% Tween 20), 0.15 mmol/L of each dNTP, 5 μmol/L of each primer, and 0.2 μL of Taq polymerase. The PCR program consisted of an initial denaturation step at 94 °C for 2 min, followed by 38 cycles comprising 20 s of denaturation at 94 °C, 30 s of annealing at 52 °C, and 40 s of extension at 72 °C. The procedure concluded with a final extension at 72 °C for 5 mins. For plastid genome sequence, short-insert (350 bp) paired-end libraries preparation and 2 × 150 bp sequencing were performed on an Illumina (Novaseq 6000) genome analyzer platform.

### ﻿Plastid genome assembly and annotation

Raw paired-end sequencing reads of the new species were filtered by the Fastp (v0.19.7) (Chen et al. 2018) to obtain high-quality clean data. The processed reads were then de novo assembled into the complete chloroplast genome using SPAdes v.3.14.1 software (Bankevich et al. 2012). Finally, it was annotated by PGA (Qu et al. 2019) with *C. urticifolia* (NC081060) as reference genome. The circular chloroplast genome map of the new species was drawn with OGDRAW (https://chlorobox.mpimp-golm.mpg.de/OGDraw.html) (Greiner et al. 2019).

### ﻿Phylogenetic analyses

For phylogenetic analyses, complete chloroplast genome sequences and ITS sequences of 10 Clematis species from subsect. Tubulosae were downloaded from the GenBank (Suppl. material 1). The chloroplast genome and ITS dataset were aligned independently using multiple alignment using fast Fourier transform (MAFFT) v7.490 implemented in Geneious Prime (Katoh and Standley 2013). Phylogenetic analyses based on ITS sequences and chloroplast genome sequences were conducted to explore the evolutionary relationship among the new species and other *Clematis* species using maximum likelihood (ML) method with MEGA 11.0 (Tamura et al. 2021). The Tamura 2-parameter model with Gamma distribution (T92+G) was employed for nucleotide substitution, with branch support assessed through 1,000 bootstrap replicates. *Anemoclema glaucifolium* (Franch.) W.T. Wang (Ranunculaceae), which exhibits significant morphological characteristics to the genus *Clematis*, serves as a stable reference for detecting ancestral traits in previous phylogenetic studies (Jiang et al. 2017, Xiao et al. 2022), and was thus chosen as an outgroup.

## ﻿Results and discussion

### ﻿Characteristics of the chloroplast genome

**Figure 1.**
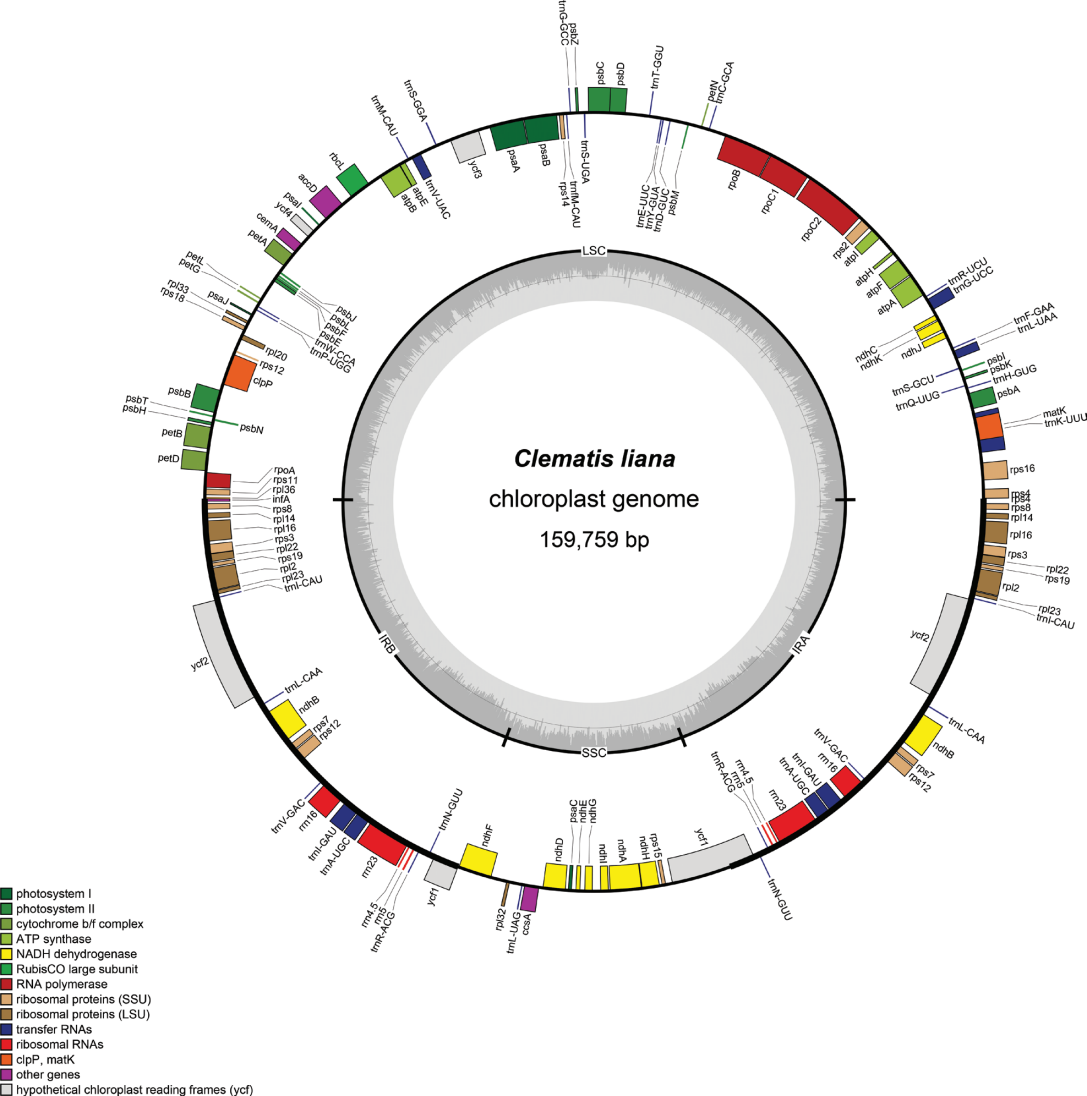
Chloroplast genome map of *Clematis liana.* The thick lines on the outer circle indicate the inverted repeat regions (IRa and IRb). The gray histogram in the innermost circle displays the GC content. Genes located in inside circle of map transcribe counterclockwise, while those outside transcribe clockwise. Various functional genes are color-coded.

The complete plastid genome sequence of *Clematis liana* sp. nov. comprises 159,759 bp, organized into a quadripartite structure containing two inverted repeat (IR: 31,084 bp each) regions, a small single copy (SSC: 18,135 bp) region, and a large single copy (LSC: 79,456 bp) region. The overall guanine and cytosine (GC) content of the plastid genome is 37.95%. The whole plastid genome contains 136 functional genes, including 92 protein-coding, 36 tRNA and 8 rRNA genes. The characteristics and statistics of plastid genome are summarized in Fig. 1 and Tables 2, 3. The complete plastid genome sequence of *C. liana* sp. nov. after annotation was deposited to GenBank under the accession No. PV335544–PV335546.

**Table 2.** Summary of whole plastid genome of *Clematis liana*.

Characteristic	Clematis liana
**Size (bp)**	159,759
**LSC length (bp)**	79456
**SSC length (bp)**	18135
**IR length (bp)**	31084
**Number of genes**	136
**Protein-coding genes**	92
**rRNA genes**	8
**tRNA genes**	36
**GC (%)**	37.95

**Table 3.** Genes encoded in plastid genome of *Clematis liana*.

Group of genes	Gene name
tRNA genes	trnA-UGC* (×*2*), *trnC-GCA*, *trnD-GUC*, *trnE-UUC*, *trnF-GAA*, *trnfM-CAU*, *trnG-GCC*, trnG-UCC*, *trnH-GUG*, *trnI-CAU* (×*2*), trnI-GAU* (×*2*), trnK-UUU*, *trnL-CAA* (×*2*), trnL-UAA*, *trnL-UAG*, *trnM-CAU*, *trnN-GUU* (×*2*), *trnP-UGG*, *trnQ-UUG*, *trnR-ACG* (×*2*), *trnR-UCU*, *trnS-GCU*, *trnS-GGA*, *trnS-UGA*, *trnT-GGU*, *trnV-GAC* (×*2*), trnV-UAC*, *trnW-CCA*, *trnY-GUA*
rRNA genes	*rrn16* (×*2*), *rrn23* (×*2*), *rrn4.5* (×*2*), *rrn5* (×*2*)
Ribosomal small subunit	*rps2*, *rps3* (×*2*), rps4*, *rps7* (×*2*), *rps8* (×*2*), *rps11*, rps12** (×*2*), *rps14*, *rps15*, rps16*, *rps18*, *rps19* (×*2*)
Ribosomal large subunit	rpl2* (×*2*), *rpl14* (×*2*), rpl16* (×*2*), *rpl20*, *rpl22* (×*2*), *rpl23* (×*2*), *rpl32*, *rpl33*, *rpl36*
DNA-dependent RNA polymerase	rpoC1*, *rpoC2*, *rpoB*, *rpoA*
Photosystem I	*psaA*, *psaB*, *psaC*, *psaI*, *psaJ*
Photosystem II	*psbA*, *psbB*, *psbC*, *psbD*, *psbE*, *psbF*, *psbH*, *psbI*, *psbJ*, *psbK*, *psbL*, *psbM*, *psbN*, *psbT*, *psbZ*
Large subunit of rubisco	*rbcL*
NADH dehydrogenase	ndhA*, ndhB* (×*2*), *ndhC*, *ndhD*, *ndhE*, *ndhF*, *ndhG*, *ndhH*, *ndhI*, *ndhJ*, *ndhK*
Cytochrome b/f complex	*petA*, petB*, petD*, *petG*, *petL*, *petN*
ATP synthase	*atpA*, *atpB*, *atpE*, atpF*, *atpH*, *atpI*
Maturase	*matK* (The *matK* is localized between the exons coding for the *trnK-UUU*)
Subunit of acetyl-CoA carboxylase	*accD*
Envelope membrane protein	*cemA*
Protease	clpP**
Translational initiation factor	*infA*
C-type cytochrome synthesis	*ccsA*
Conserved open reading frames	*ycf1* (×*2*), *ycf2* (×*2*), ycf3**, *ycf4*

Genes containing one or two introns are marked with one (*) or two (**) asterisks, respectively. Those located in the IR regions are denoted by the (× 2) symbol.

### ﻿Molecular analysis

**Figure 2.**
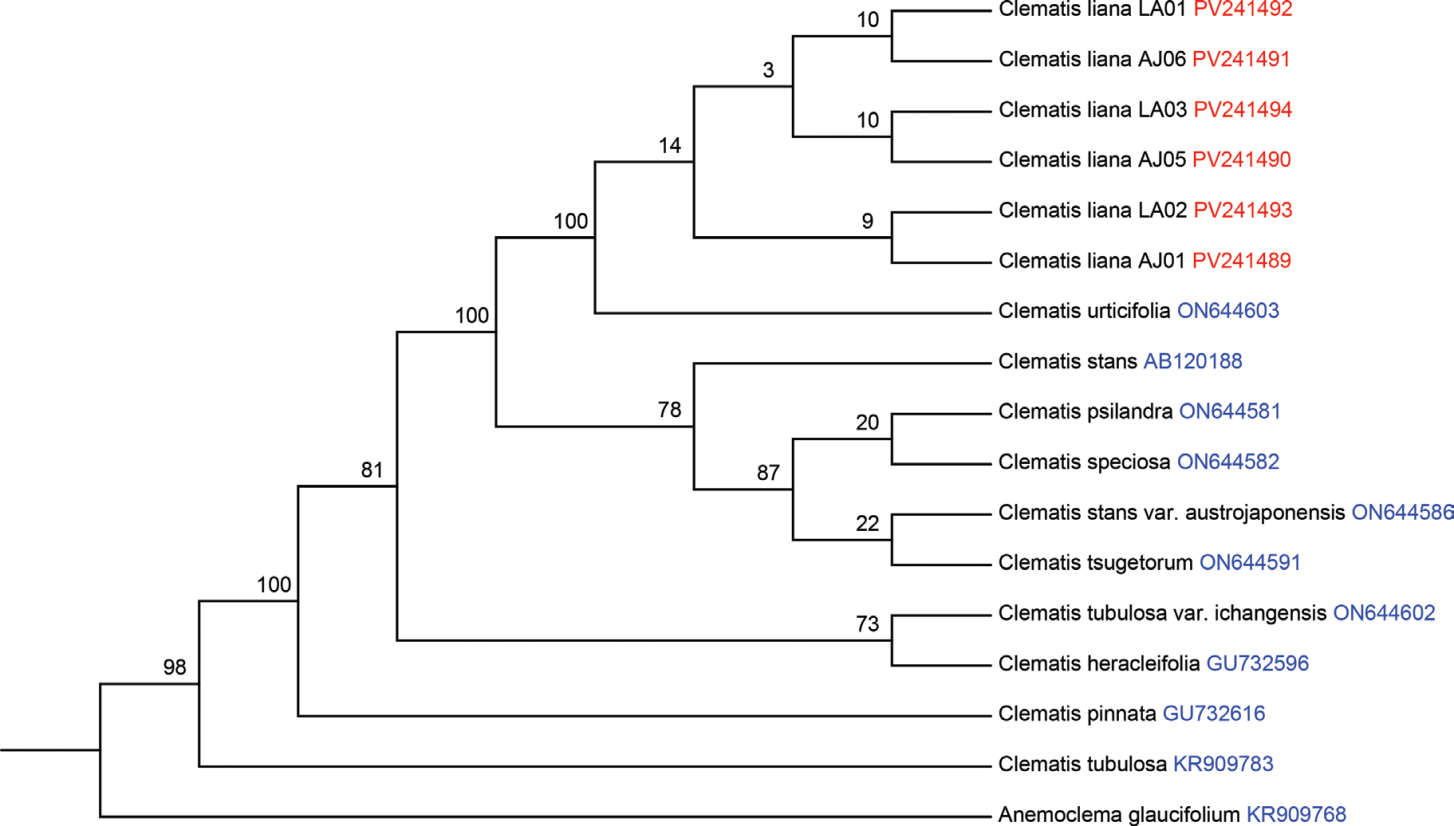
Maximum likelihood tree inferred from rDNA ITS sequences of *Clematis* to elucidate the phylogenetic position of *C. liana*

**Figure 3.**
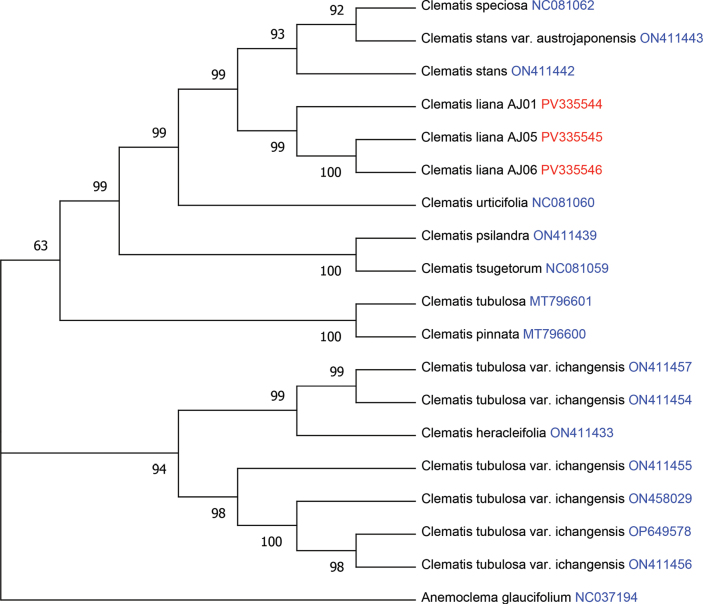
Maximum likelihood tree inferred from the chloroplast genome of *Clematis* to elucidate the phylogenetic position of *C. liana*

Phylogenetic analyses based on the plastid genome and ITS dataset consistently positioned the new species within subsection Tubulosae of the genus *Clematis*. The ITS sequences generated from our collected specimens have been deposited in GenBank under accession No. PV241489–PV241494. ML tree based on ITS data revealed that individuals of *C. liana* sp. nov. were clustered together with high bootstrap support alongside *C. urticifolia* (Fig. 2). In contrast, the ML tree based on the plastid genome data indicated that *C. liana* sp. nov. clustered with high bootstrap support within a clade comprising *C. speciosa* and C. stans var. austrojaponensis. Moreover, this clade of *C. liana* sp. nov. was further clustered with *C. urticifolia* (Fig. 3). The phylogenetic position of *C. liana* sp. nov. exhibits minor discrepancies between the plastid genome and ITS trees. Comparable inconsistencies between nuclear and plastid data have been observed in previous studies, indicating that interspecific hybridization events may be widespread in the genus *Clematis* (Xiao et al. 2022).

### ﻿Morphological comparison

**Figure 4.**
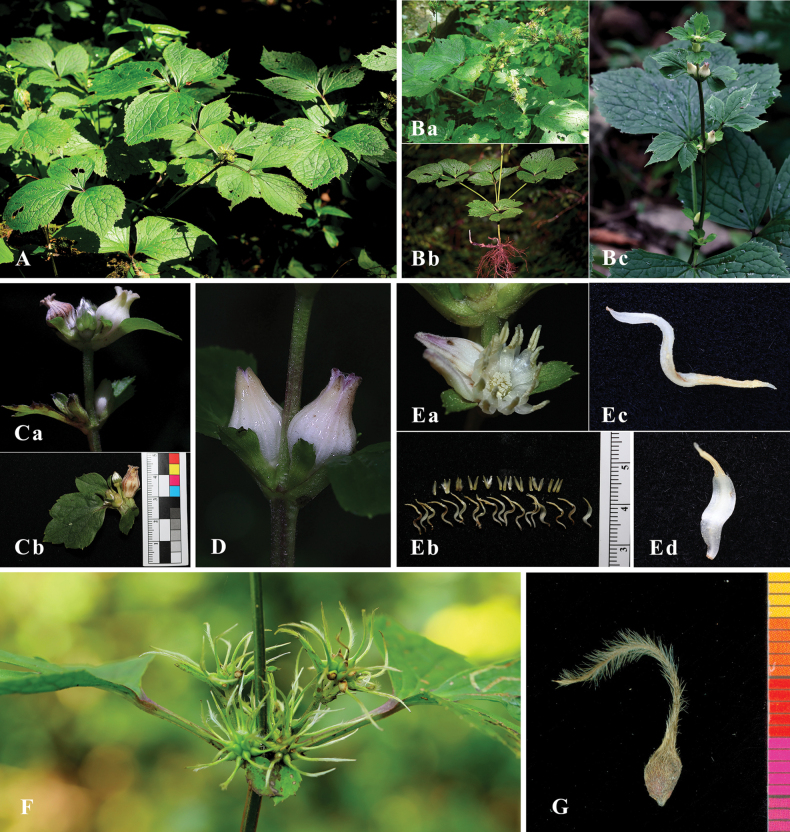
Morphological characters of *Clematis liana*. **A.** Community; **B.** Plant (**a** upper-middle part during fruiting **b** lower part showing root system and cauline leaves **c** upper part during flowering); **C.** Inflorescence (**a** upper inflorescence **b** fascicled cymose); **D.** Flower (left showing before opening; right, when open); **E.** Dissected flower (**a** stamens and pistils visible after calyx removal **b** stamens and pistils **c** stamen in lateral view **d** stamen in frontal view); **F.** Infructescence; **G.** Achene. **A. Ba** photographed by Jian-Ping Zhong in the field **Bb Bc Cb; D. Ea** photographed by Zheng-Hai Chen in the field; **G.** Photographed by Jing-Bao Pu in the lab **Ca** photographed by Jun-Feng Wang in the field **Eb Ec Ed** photographed by Jun-Ping Li in the lab; **F.** Photographed by Gen-You Li in the field

**Figure 5.**
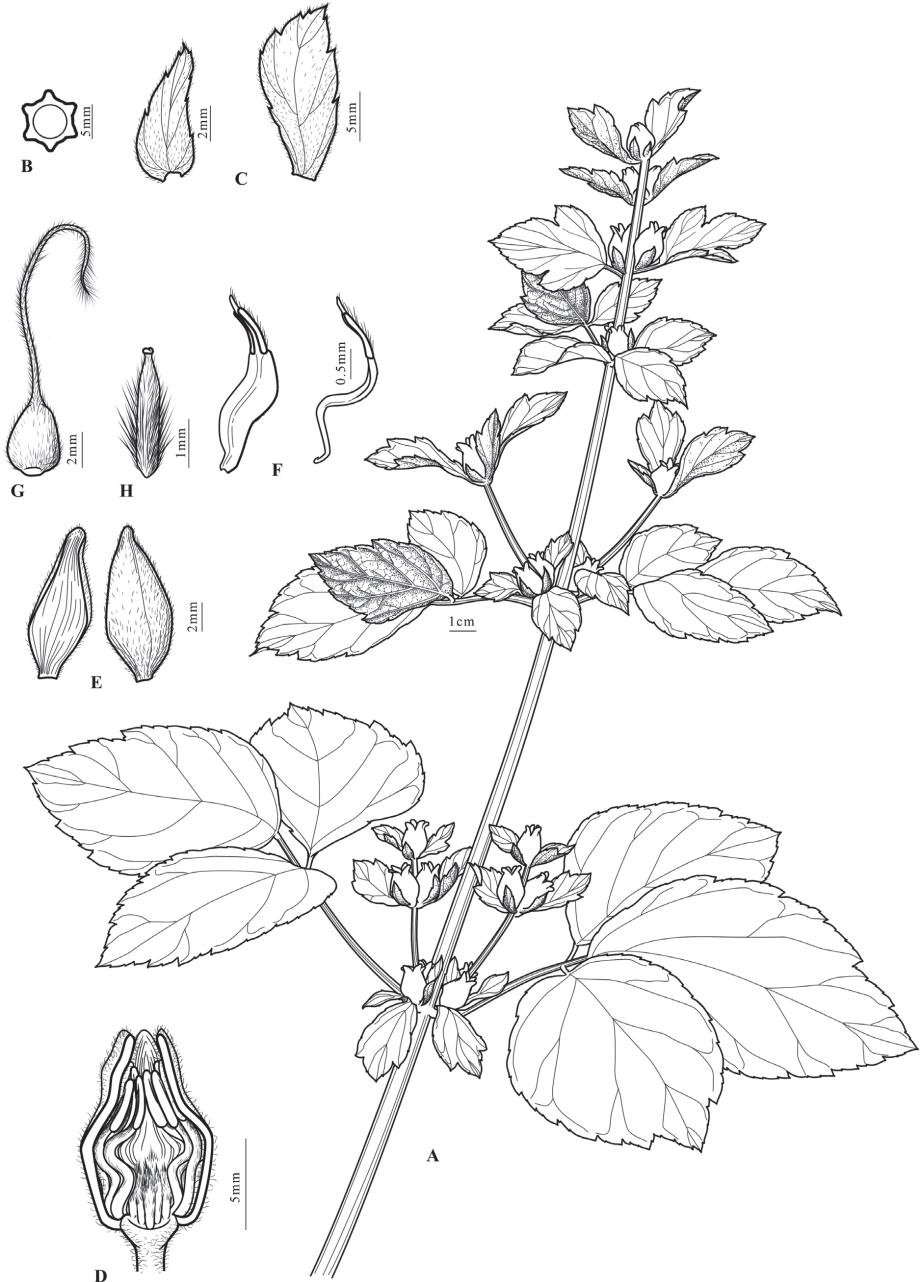
Line drawing of *Clematis liana*. **A.** Flowering branch; **B.** Cross-section of stem; **C.** Floral bract; **D.** Longitudinal section of flower (showing stamens & pistils); **E.** Sepal (inner surface & outer surface); **F.** Stamen (inner whorl & outer whorl); **G.** Achene with persistent style; **H.** Lateral view of achene. Drawn by Yi Zhang, based on *Jun-Ping Li*, *Xian-Ting Wang*, *Zheng-Hai Chen*, *Wei-Qing Liang* et al. *TXLAJ2024080501* (ZM, barcode NH0067429!)

The morphological characteristics of the new species, such as lifeform, stem, leaflet blade, inflorescence, flowers, sepal, and stamen were comprehensively studied and compared with related species, and the results showed that the new species is clearly different from other related species (Figs 4, 5).

The distinguishing characteristics of the new species and the three relatives in subsect. Tubulosae are listed in detail in Table 4. The morphological analysis reveals that although *C. liana* sp. nov., *C. urticifolia*, *C. satomiana*, and C. stans var. austrojaponensis share similarities in certain traits, they exhibit distinct characteristics. Compared to *C. liana* sp. nov., *C. urticifolia* is a subshrub with polygamous and purple flowers, an urceolate or tubular-urceolate calyx, 12–16 stamens, lanceolate-linear filaments that are 7–9 mm long and pilose near the apex, linear anthers that are 4–5 mm long, and ca. 12 carpels. Unlike *C. liana* sp. nov., *C. satomiana* is a subshrub with dioecious, pale violet flowers, featuring a tubular-urceolate or tubular calyx that is 18–20 mm long, strongly recurved sepals with sagittate and slightly dilated apical parts, and filaments that are 3 mm long, shorter than the anthers and glabrous. Moreover, C. stans var. austrojaponensis differs from *C. liana* sp. nov. in that it is a subshrub with dioecious, pale violet or pale purplish, nodding flowers, featuring a tubular-urceolate calyx 1.0–2.6 mm long and sepals with apices strongly recurved to 180 degrees.

**Table 4.** Morphological comparison between *Clematis liana* and its allied species.

Characters	*C. liana* ^δ^	*C. urticifolia* ^β,γ^	*C. satomiana* ^α^	C. stans var. austrojaponensis ^α,γ^
Lifeform	perennial herb	subshrub	deciduous subshrub	deciduous subshrub
Reproductive strategy	hermaphroditic	polygamous	dioecious	polygamous
Stem	erect, 0.5–1.0 m tall, sharply 6-ridged or narrowly winged, deeply 6-sulcated, initially sparsely white puberulous, glabrescent	erect, up to 2 m tall or more, longitudinally sulcate, puberulous, lower part woody	erect, 0.5–2.0 m tall, to 3 cm in diam. at base, sulcate, sparingly strigose, lower part woody	erect or sometimes ascending, up to 1 m tall, sulcate, sparingly strigose or glabrous, lower part woody
petiole	up to 20 cm long	8.5–16.3 cm long	1.5–8.0 cm long, densely tomentose, not tendrilous	4–20 cm long, strigose, not tendrilous
Terminal leaflet blade	chartaceous, broadly ovate, ovate-circular to suborbicular, 8–14 × 5–14 cm, shallowly 3-lobed or undivided apically, apex acute to shortly-acuminate, base rounded or cuneate, adaxially subglabrous, abaxially sparsely pubescent along veins, margin irregularly dentate, teeth tip short-pointed	papery, broadly rhombic, rhombic, broadly ovate, elliptic, or obovate, 3-lobed or 3-lobulate, 7–16 × 4–11 cm, apex acuminate, base broadly cuneate or rounded, adaxially appressed-puberulous, abaxially reticulate, pubescent, margin irregularly dentate	coriaceous, obovate to narrowly obovate, sometimes ovate or widely lanceolate, shallowly trilobed, 9–15 × 5–8.5 cm, apex acuminate to acute, base cuneate to rounded, abaxially tomentose along veins, margin coarsely serrate	coriaceous, widely ovate, medially trilobed, 5–14 × 3–11 cm wide, apex acuminate, base rounded to widely cuneate or subtruncate, abaxially sericeous, tomentose along veins, margin coarsely serrate
Lateral leaflet blade	smaller, asymmetrical	smaller, obliquely ovate	ovate to lanceolate, almost simple, coarsely serrate	/
Inflorescence	cymose 1–3-flowered, fasciculate, axillary	panicles terminal, 3–40 cm long, 2–3 times branched, 10–many-flowered	compound cymose terminal and axillary, 3–20 cm long, flowers many	compound cymose, terminal ones 5–16 cm long, axillary ones 4–10 cm long, shorter than leaves, flowers many
Involucral bract	ovate to oblanceolate, asymmetrical, apex 2- or 3-teeth, 12–15 mm long, puberulous	foliaceous or simple, obovate	trilobed to simple and lanceolate, sometimes large and foliaceous, 10 × 10 cm	trifid to simple
Floral bract	narrowly ovate to narrowly ovate-lanceolate, 5–8 mm long, puberulous	linear-lanceolate or triangular, 1.2–5 mm long, densely puberulous	/	/
Pedicel	stout, 2–9 mm,1.5–1.7 mm in diam., short pubescence	robust, 0.5–3 mm long, 1.2–1.8 mm in diam., velutinous	8–12 mm long, sericeous-tomentose	3–10 mm long, densely tomentose
Flower	urceolate, ca. 1.8 cm in diam., fragrant	urceolate or tubular-urceolate, 8 mm in diam., often 3-costate, violet, glabrous inside, appressed-velutinous outside, velutinous on margin	tubular-urceolate (♀), or tubular (♂), 18–20 × 3 mm, pale violet, erect to oblique or horizontal, abaxial surface sericeous-tomentose,	tubular-urceolate (♀), 1–2 cm long, or urceolate (♂), 8-16 mm long, pale purplish, nodding
Sepal	creamy white or pale yellow with pale purple apices, narrowly ovate or lanceolate, ca. 1.5 × 5.5 mm, apex acuminate, recurved outward	purple, oblong-lanceolate or narrowly ovate, 12–15 × 3–6 mm, apex acuminate, recurved	strongly recurved, with sagittate and slightly dilated apical parts, connate at 1–9 mm from base (♀), or connate at 2–7 mm from base or free (♂)	apex attenuated and gently recurved to 180 degrees, connate at 7–12 mm from base (♀), or slightly dilated and strongly recurved to 360 degrees or more, connate at 2–5 mm from base or almost free (♂)
Stamen	16–18, 9–11 mm long; filaments 5–6 mm long, glabrous; anthers 2.9–3.6 mm long; connective sparsely pilose, apex finely pointed	12–16, 9–13 mm long; filaments lanceolate-linear, 7–9 mm long, 1-veined, pilose near apex; anthers 4–5 mm long, linear, apex apiculate; connective very developed, with a purple top	ca. 8 mm long (♂), or ca. 5 mm long, sterile (♀); filaments 3 mm long, shorter than anthers, glabrous (♂); anthers 5 mm long, linear-ovate (♂); connective sericeous-villose, with exserted tips 0.5 mm long (♂)	filaments 2–3 times longer than the anthers, glabrous; anthers 3–4 mm long, linear-ovate; connective sericeous-villose, with exserted tips ca. 1 mm long (♂)
Pollen grains	pantoporate, 25.0 μm in diam.	pantoporate, 21.8 μm in diam.	/	pantoporate, 21.4 μm in diam.
Carpel	19–20, 2.5 –3.0 mm long, densely white sericeous-villous	ca. 12, 4 mm long, densely villous	style ca. 1 cm long, plumose (♀), or abortive, sterile (♂)	/
Achene	slightly compressed, ovoid, 3.1–4.2 × 2.2–3.7 mm, reddish-brown, sparsely appressed-puberulous; persistent styles 1.1–2.5 cm long, plumose.	compressed, broadly ovate or broadly elliptic, 3–3.2 × 2.8–3.2 mm, pilose, not or slightly rimmed; persistent style ca. 2.5 cm long, plumose	ovoid, 3–4 mm long, light reddish brown, hirsute; persistent style 16–20 mm long, yellowish, plumose	obovoid, 2–3.5 mm long, dark brown, hirsute; persistent style 15–20 mm long, yellowish, plumose
Phenology	flowering late July-August, fruiting late October-November	/	flowering from August to September	flowering from August to October
Habitat	in gravel accumulations under deciduous broad-leaved forests; alt. 640–1,463 m a.s.l.	at forest edges or in sparse forests on slopes; alt. 700–1,300 m a.s.l.	beside and in summer-green forests	beside and in summer green forests in calcareous areas; 200–1,500 m a.s.l.
Distribution	China: Zhejiang and Anhui	S Korea	Japan: Honshu	Japan: Shikoku and Kyushu

Based on Kadota 2006α, Kitagawa 1937β, Wang and Xie 2007γ; and own measurements at ZMδ. “/” represents data deficient.

### ﻿Taxonomic treatment

#### ﻿ Clematis liana

Taxon classificationPlantaeRanunculalesRanunculaceae

Z. H. Chen, J.P. Li, J.B. Pu et W.Y. Xie sp. nov.

4AA1A088-C224-534D-A071-5B045867AE23

urn:lsid:ipni.org:names:77372763-1

**Figure 6.**
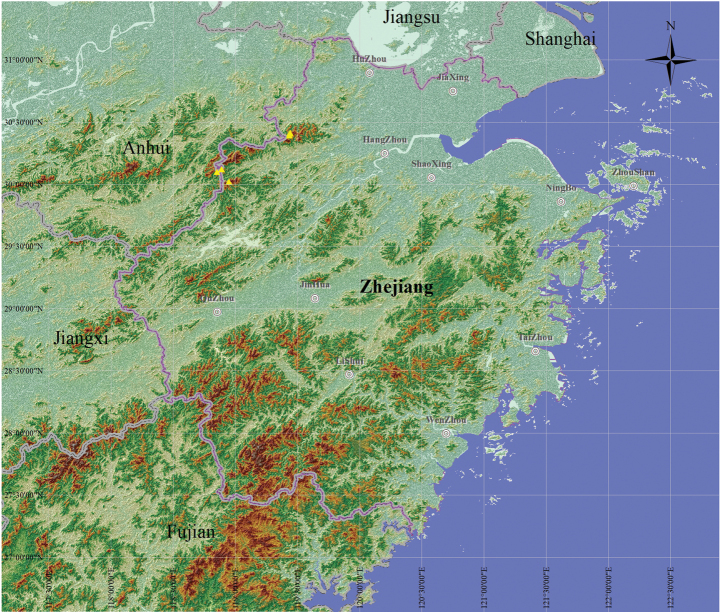
Geographic distribution of *Clematis liana* in Zhejiang Province and Anhui Province (yellow triangles).

Figs 4, 5, 6 Chinese name: 安吉铁线莲 (Ān Jí Tiě Xiàn Lián)

##### Type.

China • Zhejiang Province: Huzhou City, Anji County, Longwangshan mountain, Dongguan to Xianrenqiao, shaded valley under deciduous broad-leaved forest, 30°24'25.55"N, 119°26'35.69"E, alt. 1,248 m a.s.l., 5 August 2024, *Jun-Ping Li*, *Xian-Ting Wang*, *Zheng-Hai Chen*, *Wei-Qing Liang et al.* TXLAJ2024080501 (holotype: ZM [barcode NH0067429!]; isotypes: ZM [barcode NH0067430!], ZJMR [barcode 00015501!]).

##### Diagnosis.

*C. liana* sp. nov. is morphologically similar to *C. urticifolia* Nakai ex Kitag., but differs by the following characters: perennial herb with hermaphroditic flowers (vs. subshrub with polygamous flowers); floral bracts narrowly ovate to narrowly ovate-lanceolate, subentire, 5–8 mm long (vs. linear-lanceolate or triangular, 1.2–5 mm long); flowers bisexual, urceolate (vs. urceolate or tubular-urceolate); calyx creamy white or pale yellow with pale purple apices (vs. entirely purple); stamens 16–18 (vs. 12–16), filaments narrowly oblong, 5–6 mm long, glabrous (vs. lanceolate-linear, 7–9 mm long, pilose apically), anthers narrowly lanceolate, 2.9–3.6 mm long (vs. linear, 4–5 mm long), pollen grain 25.0 μm in diam. (vs. 21.8 μm); carpels 19–20 (vs. ca. 12); stems and petioles sharply longitudinal-edged or narrowly winged (vs. blunt longitudinal-ridged); leaflet blade broadly ovate, ovate-circular to subcircular, apex shortly acuminate (vs. broadly rhombic to obovate, apex acuminate).

##### Description.

***Perennial herb***, 0.5–1.0 m tall. ***Roots*** woody, brownish. ***Stems*** erect, robust, green, sharply 6-ridged or narrowly winged, deeply 6-sulcated, initially sparsely white-puberulent, glabrescent at maturity. ***Leaves*** ternate (upper leaves simple, trilobate to subtrilobate); petioles stout, up to 20 cm long, adaxially deeply sulcated, abaxially sharply-ridged, basally slightly widened and connate to opposite petiole, often tinged purple, initially sparsely pubescent, glabrescent at maturity; terminal leaflets chartaceous, broadly ovate to suborbicular, 8–14 × 5–14 cm, shallowly 3-lobed or undivided apically, apex acute to shortly acuminate, base rounded or cuneate, adaxially green, subglabrous (veins impressed), abaxially pale green (veins prominent, sparsely pubescent along veins), margin irregularly dentate (teeth tip short-pointed), petiolules 3–4 cm long, often tinged purple; lateral leaflets smaller, asymmetrical, petiolules 3–8 mm long, base tinged purple. ***Inflorescence*** cymose 1–3-flowered, fasciculate, axillary, subsessile; involucral bracts ovate to oblanceolate, asymmetrical, apex 2- or 3-dentate, 12–15 mm long (including stalk), puberulous; floral bracts narrowly ovate to narrowly ovate-lanceolate, subentire, puberulous, 5–8 mm (including stalk); stalk adaxially base often tinged purple. ***Flowers*** bisexual, urceolate, ca. 1.8 cm in diam., fragrant; pedicels stout, 2–9 mm long, 1.5–1.7 mm in diam., short-pubscent; sepals 4, imbricate, erect, creamy white or pale yellow with pale purple apices, narrowly ovate or lanceolate, ca. 15 × 5.5 mm, apex acuminate, recurved outward, outer surface densely appressed-puberulent (velutinous on margins), inner surface glabrous, 3-veined; stamens 16–18, 9–11 mm; filaments narrowly oblong, S-shaped, 5–6 mm, white, glabrous; anthers narrowly lanceolate, 2.9–3.6 mm, connective sparsely pilose, apex finely pointed; pollen pantoporate, 25.0 μm in diam. Carpels 19–20, 2.5–3.0 mm, densely white sericeous-villous. ***Achenes*** slightly compressed, ovoid, 3.1– 4.2 × 2.2–3.7 mm, reddish-brown, sparsely appressed-puberulous; persistent styles 1.1–2.5 cm, plumose.

##### Phenology.

Flowering late July–August; fruiting late October–November.

##### Etymology.

The species epithet *liana* honors Professor Li Gen-you (李根有), a renowned botanist at Zhejiang A&F University, who first recognized the taxonomic distinctiveness of this species during field surveys in 2013.

##### Distribution and ecology.

*Clematis liana* is currently known from Anji County (Huzhou City) and Lin’an District (Hangzhou City) in Zhejiang Province, and She County (Huangshan City) in Anhui Province, eastern China. It grows in gravel accumulations under deciduous broad-leaved forests within valleys or on mid-elevation mountain slopes, at altitudes of 640–1,463 m.

##### Conservation assessment.

We calculated the conservation metrics for *Clematis liana* sp. nov. using GeoCAT (Bachman et al. 2011), with an Area of Occupancy (AOO) of 24.0 km^2^ and Extent of Occurrence (EOO) of 442.7 km^2^, based on field investigations and herbarium specimen records. The five known subpopulations collectively contain fewer than 1,000 mature individuals. Based on IUCN (2024) Endangered (EN-C2) *C. liana* sp. nov. is assessed as **Endangered (EN)**.

##### Additional specimen examined (Paratypes).

**China** • **Zhejiang Province**, Huzhou City, Anji County, Longwangshan mountain: Xianrenqiao, understory of valley forests alt. 900 m, 24 August 1988, *Yue-Liang Xu* & *Fang-Gang Zhang 0600* (ZM); • ibid., Qianmutian trail, forested streamside gravel, alt. 920 m a.s.l., 28 July 2015, *Xiao-Feng Jin s.n.* (PE); • ibid., understory of valley forests, 30°24'27.58"N, 119°26'28.71"E, alt. 1,173 m a.s.l., 5 August 2024, *Jin-Bao Pu*, *Zheng-Hai Chen*, *Jun-Ping Li*, *Li-Peng Yu et al. TXLAJ2024080505* (ZM); • ibid., understory of valley forests, 30°24'29.46"N, 119°26'22.52"E, alt. 987 m a.s.l., 5 August 2024, *Jin-Bao Pu*, *Zheng-Hai Chen*, *Jun-Ping Li*, *Wei-Qing Liang et al. TXLAJ2024080506* (ZM). • Hangzhou City, Lin’an District, Xitianmushan mountain, Pingxi, 17 October 1952, *Xian-Yu He New1017* (IBSC, NAS); • ibid., Sandaoling, in damp and shady places under deciduous broad-leaved forest, 30°23'35.39"N, 119°26'11.41"E, alt. 1,463 m a.s.l., 26 September 2024, *Fen-Yao Zhang & Qi-Biao Xu 2024092601* (ZM); • Qingliangfeng mountain, Shunxiwu, Taoshuwan, 30°1'10"N, 118°56'24"E, alt. 1,028 m a.s.l., 10 November 2024, *Wei-Qing Liang LATXL2024111001, LATXL2024111002, LATXL2024111003* (ZM); • ibid., Kuliwan, grassy streamside, alt. 640 m a.s.l., 16 Sep. 1957, *Xian-Yu He 23934* (IBSC, NAS, PE); • ibid., Zhaojiakeng, under forest by creek in south-west valley, 24 Sep. 1958, *Xian-Yu He 30502* (IBSC, PE); Qingliangfeng, Niulangping, 30°7'16.38"N, 118°53'26.13"E, alt. 1,290 m a.s.l., 30 October 2023, *Wen-Yuan Xie s.n.* (achenes, ZJFC). • **Anhui Province**, Huangshan City, Shexian County, Sanyang Town, Qingliangfeng, Laoguaidui, beside ditch in valley, 30°5'56.41"N, 118°51'12.50"E, alt. 980 m a.s.l., 31 August 1983, *Xiao-Ping Zhang 0931* (IBSC).

## Supplementary Material

XML Treatment for Clematis liana

## ﻿Additional information

### Use of AI

No use of AI was reported.

### Author ORCIDs

Pan Xu https://orcid.org/0000-0002-3309-773X

Jun-Ping Li https://orcid.org/0009-0000-7655-3849

Xian-Ting Wang https://orcid.org/0009-0009-6044-5857

Fen-Yao Zhang https://orcid.org/0000-0001-5695-0230

Wei-Qing Liang https://orcid.org/0009-0003-2948-0845

Wen-Yuan Xie https://orcid.org/0000-0003-1529-0787

Li-Peng Yu https://orcid.org/0009-0004-6503-9034

Feng Chen https://orcid.org/0000-0002-3560-6710

Ke-Lang Lou https://orcid.org/0009-0006-7095-9163

Jian-Ping Zhong https://orcid.org/0009-0008-3091-8791

Jin-Bao Pu https://orcid.org/0000-0003-4911-1169

Zheng-Hai Chen https://orcid.org/0000-0002-8852-7127

### Data availability

All of the data that support the findings of this study are available in the main text or Supplementary Information.

## Supplementary materials

Supplementary material 1 NCBI accessions of studied samples Data type docx https://binary.pensoft.net/file/1483305This dataset is made available under the Open Database License (http://opendatacommons.org/licenses/odbl/1.0/). The Open Database License (ODbL) is a license agreement intended to allow users to freely share, modify, and use this Dataset while maintaining this same freedom for others, provided that the original source and author(s) are credited.Pan Xu, Jun-Ping Li, Xian-Ting Wang, Fen-Yao Zhang, Wei-Qing Liang, Wen-Yuan Xie, Li-Peng Yu, Feng Chen, Ke-Lang Lou, Jian-Ping Zhong, Jin-Bao Pu, Zheng-Hai Chen
