# Supplementary material for: ﻿A new species of Clematis sect. Tubulosae (Ranunculaceae) from Zhejiang, East China
Source: PhytoKeys. 2025 Dec 3;267:93–108. doi: 10.3897/phytokeys.267.158140 (PMC12696475; doi:10.3897/phytokeys.267.158140)
Supplement: Supplementary material 1 — NCBI accessions of studied samples [file phytokeys-267-093_article-158140__-s001.docx]

**Supplementary Table 1. NCBI accessions of studied samples**

| **Taxon** | **ITS Accession No.** | **References** | **Chloroplast genome Accession No.** | **References** |
| --- | --- | --- | --- | --- |
| *Clematis urticifolia* Nakai ex Kitag. | ON644603 | Lyu et al.2023 | NC081060 | Lyu et al.2023 |
| *Clematis stans* Siebold & Zucc. | AB120188 | Miikeda et al. 2006 | ON411442 | Xiao et al.2022 |
| *Clematis psilandra* Kitag. | ON644581 | Lyu et al.2023 | ON411439 | Xiao et al.2022 |
| *Clematis speciosa* (Makino) Makino | ON644582 | Lyu et al.2023 | NC081062 | Lyu et al.2023 |
| *Clematis stans* var. *austrojaponensis* (Ohwi) Ohwi | ON644586 | Lyu et al.2023 | ON411443 | Xiao et al.2022 |
| *Clematis tsugetorum* Ohwi | ON644591 | Lyu et al.2023 | NC081059 | Lyu et al.2023 |
| *Clematis tubulosa* var. *ichangensis* (Rehder & E.H.Wilson) W.T. Wang | ON644602 | Lyu et al.2023 | ON411457, ON411454,  ON411455, ON458029,  OP649578, ON411456 | Xiao et al.2022 |
| *Clematis heracleifolia* DC. | GU732596 | Xie et al. 2011 | ON411433 | Xiao et al.2022 |
| *Clematis pinnata* Maxim. | GU732616 | Xie et al. 2011 | MT796600 | Lyu et al.2021 |
| *Clematis tubulosa* Turcz. | KR909783 | Lehtonen et al. 2016 | MT796601 | Lyu et al.2021 |
| *Anemoclema glaucifolium* (Franch.) W. T. Wang | KR909768 | Jiang et al. 2017 | NC037194 | Jiang et al. 2017 |
